# Historical Sketch of the Medical Society of the County of Erie—1821–18961Read by appointment at the annual meeting of the society, January 14, 1896.

**Published:** 1896-02

**Authors:** Franklin C. Gram

**Affiliations:** Secretary


### HISTORICAL SKETCH OF THE MEDICAL SOCIETY OF THE COUNTY OF ERIE.[1]

### 1821–1896.

BY FRANKLIN C. GRAM, M. D., Secretary.

WHEN a society has existed long enough to celebrate its seventy-fifth anniversary, it certainly becomes of interest to others than those associated with it. The Medical Society of the County of Erie has the honor of celebrating this event today. It deserves recognition, inasmuch as the history of this society also involves considerable of the history of Western New York.

1. Read by appointment at the annual meeting of the society, January 14, 1896.

It is unfortunate that the value of records was so little appreciated, and, furthermore, that through the many changes of administration the most valuable portions of the early records have been lost. Most of those still in existence are in the shape of reports written on scraps of paper, and by going through a chaotic mass of these—sufficient in quantity to fill several large baskets—I was enabled to make some kind of a connected story from the birth of the society to the present time.[2]

At the time of the society's organisation, Buffalo was but a village. One of the most prominent residents, as well as the foremost in his profession, was Dr. Cyrenius Chapin. He had an office on Swan street, on the site now occupied by the Chapin Block. As far as is known, no portrait of him is in existence, although on several occasions diligent search has been made for one. Dr. Chapin came from Massachusetts to Buffalo in 1805, when the place, which was then called New Amsterdam by Ellicott, had about a dozen dwellings. He took a prominent part in the war of 1812 and was surgeon to the military hospital. Later he became president of the county agricultural society and a trustee of the village. He died in 1838, at the age of 69 years.

When Dr. Chapin came here this was still Niagara county and a medical society was organised in 1808, thus giving that county priority in date. About the year 1820 the county was divided, thereby creating Erie county, and this gave Dr. Chapin an opportunity to organise a society in the new county. The first meeting was held at the house of P. M. Pomeroy, in the village of Buffalo, January 9, 1821, when an organisation was effected with the following officers: president, Dr. Cyrenius Chapin; vice-president, Dr. Daniel Chapin; secretary, Dr. John E. Marshall; treasurer, Dr. Lucius H. Allen; censors, Drs. Chas. Pringle, Sylvanus S. Stewart, Benjamin C. Congdon, L. H. Allen and J. E. Marshall.

The remaining charter members, making twenty-four in all, were: Drs. Ebenezer Johnson, Daniel Allen, Jonathan Hoyt, Daniel Ingalls, Charles McLowth, Josiah Trowbridge, Elisha Smith, Sylvester Clark, Jonathan Hurlburt, Rufus Smith, Ira G. Watson, Varney Ingalls, William H. Pratt, William Lucas, John Watson, Thos. B. Clark and Dr. Woodward.

Niagara county was represented in the state society in 1817 by Dr. James H. Richardson, and Erie county in 1823 by Dr. L. H. Allen.

---

2. Since this article was written, a book supposed to be lost, containing the minutes from 1834 to 1871, has been discovered and is again in the possession of the society.

At the first meeting, Dr. Chapin delivered an address in which he, at that early date, inveighed against quacks, who did no end of harm, and also deplored the "strange inconsistency and cold ingratitude of the public toward the medical profession," and further, that "the truth is too obvious to require illustration, that the profession is far from maintaining the rank among the learned professions, which its consequence demands." He then called attention to the fact that a physician's services were undervalued by the public, and as they were not charitable institutions it was time to resolutely determine upon a total reformation.

In a "public notice," which he issued about that period, he felt it his duty to inform those indebted to him for professional services, that the time had arrived when imperious necessity compelled him to an immediate collection of his demands. Continuing, he says:

It has too long been a prevalent idea with the public that a physician's bill is never to be paid; and to call upon a patient when restored to health and to the enjoyments of life through the skill and attention of his physician, for a reward for the services rendered, is considered almost an insult and certainly a hardship. . . . . If there is a class of men in the community entitled to liberal compensation and prompt pay for their services, certainly physicians are. The enjoyment of health is first upon the catalogue of life's blessings and without it every other enjoyment is embittered. . . . . As a matter of self-preservation they must collect their debts, the people must reward them for their services, and the idea that they are not at liberty, from the general sense of the public on the subject, to force collections, as do other professions, but must drag out their lives in penury and distress, is both unjust and humiliating. To relieve my own necessities I am compelled to resort to immediate collections, and this I shall do without discrimination. Those, therefore, who feel it a privilege to save the costs of prosecution will find it expedient to bestow immediate attention to this subject.

Buffalo is under obligations to Dr. Daniel Chapin, who came from Connecticut in 1806, and settled on a farm in the locality which now comprises the park meadow and neighboring groves. Many of the trees which beautify that place were planted by him. He was noted for his antagonism to his namesake, claimed to be president of the Niagara county society, and died in 1821.

Dr. Ebenezer Johnson, whose name appears second on the list, but who, at that time, was about to retire from professional life, came to Buffalo in 1809, and served as surgeon in the War of 1812.

He subsequently went into business and became the first mayor of Buffalo. He built a stone mansion on Delaware avenue, which later became a residence connected with the Female Academy, and still stands.

Dr. Josiah Trowbridge, likewise a native of Connecticut, came here in 1811. Buffalo was too slow for him, so he went to Fort Erie, where he practised until the breaking out of the war, when his patriotic spirit induced him to return. He was president of the society in 1839, and its librarian from 1843 to 1853. He started the agitation which finally resulted in a law providing dissecting material through unclaimed bodies of persons dying in public institutions. He was the first president of the Buffalo Medical Association in 1845, was supervisor for several years, judge of the Court of Common Pleas, and finally mayor of Buffalo in 1837. He died in 1862.

Dr. John E. Marshall, another Connecticut man, was the first physician to settle in Mayville, and became clerk of Chautauqua county at its organisation in 1811. He was surgeon to McMahon's regiment during the war of 1812, and came to Buffalo in 1815. He was clerk of Niagara county in 1818, treasurer of the Erie county medical society from 1826 to 1828, its president in 1830, and the first health physician of the City of Buffalo in 1832. He died in 1838.

Dr. Jonathan Hoyt resided in Aurora, was judge of the county for several years, and died in 1850. Dr. Ira G. Watson settled in South Wales in 1812, residing there until his death in 1847, enjoying an extensive practice in the towns of Wales, Aurora, Holland and Colden. Dr. John Watson was the first physician in Aurora, in 1811; Drs. Daniel and Varney Ingalls in Springville, in 1818, and Dr. Wm. H. Pratt in Eden. Dr. Bela H. Colegrove came to Sardinia from Rhode Island, in 1820. He was president of the society in 1828 and died in 1874, having enjoyed during that period the reputation of being the most noted surgeon in this and the three surrounding counties.

Dr. Colegrove also became noted as the first physician to successfully antagonise the rules of the county medical society. This organisation, by the way, possessed very extraordinary privileges and was in turn compelled to follow certain rules regardless of the wishes of its members. No person was legally entitled to practise medicine or surgery in this county, except he was a member of this society. That made it compulsory for him to join, and, if otherwise qualified, the society had no right to refuse him admission. On one occasion the majority thought differently and it cost the society about $600 to learn this fact. Likewise there was virtually no way of getting out and the society could exercise all sorts of discipline. One of the society's hobbies was to secure the attendance of its members at its meetings, or die in the attempt, and to accomplish that end a by-law was adopted imposing a penalty of $1.00 for absence at any meeting. That this rule was not intended as a dead letter is shown by the following resolution, adopted at one of the sessions: "Resolved, that the treasurer be directed to collect outstanding dues from members—peaceably if he can, forcibly if he must."

A large bundle of letters sent in response to an effort of the treasurer, under this resolution, still exists, and indicates the unpopularity of the proceedings. Most of these letters are interesting reading, but one of them was evidently intended for a place in modern literature, even at that period, for it contains the indorsement, "admirable"—presumably inscribed by one of the officers. It was written by Dr. Colegrove to Dr. Marshall, and dated Sardinia, June 11, 1838. In it Dr. Colegrove protests against paying fines for non-attendance because the rule is unjust and discriminates against members. He said that he lived thirty miles from the place where the meetings are required by law to be held, and to go there twice a year meant a sacrifice to him of some $15 or $20, or pay a penalty, which the city members could avoid by the sacrifice of so many pence! He did not think he ought to compromise the interests of the community where he resided through neglect caused by attending the meetings of a medical society! "It is not the amount," said he, "at which I complain, but the principle, and I would as soon—and with equal justice—make the penalty for non-attendance 'imprisonment in the county jail for the term of six months,' as to have it as it now is."

Dr. Luther Spaulding, of Williamsville, became sarcastic when asked for $7.00 in fines and wanted to know if there was not some mistake about the entire affair and that he ought to be charged with fines for ten years more. Another doctor said there was already one judgment against him and the society was welcome to the second! Dr. W. H. Pratt, of Eden, presented his compliments and said he possessed only a small share of the good things of this world; he had never attended the society's meetings and could not now, owing to physical debility—but they appear to have kept on fining him just the same.

Dr. Austin Flint, the founder of the BUFFALO MEDICAL JOURNAL, DR. F. H. Hamilton, Dr. James P. White and Dr. John C. Dalton—all men with more than a national reputation—fared no better. In the ledger, Dr. Flint is charged with $1.00 tax, or dues, and only $4.00 fines to begin with. The same proportion continues throughout his entire account.

This ledger, by the way, is another curiosity, and is the only remnant of the earlier days which makes any attempt at regularity. It was evidently in the hands of a systematic bookkeeper for a number of years. Whenever the debtor side got too large he had the fines remitted by resolution, so as to balance the account. In Dr. Gilbert McBeth's account he is credited with "Death by cholera, $6.00,"—whatever that meant,—and this was afterward remitted by the society to force a balance.

The first treasurer's report obtainable is dated January 9, 1827, and is signed by Dr. Marshall. It shows that the receipts for the previous year were $11.00 and the disbursements $8.00. At that time there were twenty-four books in the library. In 1830 the treasury contained six shillings, while the debts amounted to $10.50. The receipts did not grow very much for the next thirty years, until in 1858 the society was well enough situated to incur $52.00 legal expenses for prosecuting one of its members, and lost its case at that.

The society appears to have been among the first in the State to appreciate the value of vital statistics and drafted a bill which was sent to the legislature. It also devoted much attention to the subject of vaccination, and was always aggressive in the various branches of medical science. This society is also mainly responsible for the present higher medical education law, as well as the one to regulate the practice of medicine, and to Dr. Edward Storck undoubtedly belongs the credit, in a large measure, of bringing this question to a successful issue.

Many physicians of note, during the earlier and later periods, can only be given a passing mention. Among them are Dr. Bryant Burwell, who came to Buffalo in 1824, was associated for a time with Dr. Chapin, and later held, perhaps, more positions of note than any of his medical contemporaries. His active career ended in 1862. Dr. Alden S. Sprague, 1825–1863; Dr. Charles Winne, a prominent surgeon, 1833–1877; Dr. Gorham F. Pratt;

Dr. George Burwell; Drs. H. B. Camp and G. H. Lapham, of Aurora; Dr. J. E. Hawley; Drs. I. H. Hopkins and Young, of Tonawanda; Dr. B. A. Batty, of Boston; Dr. Harvey H. Hubbard, of Springville; Dr. N. D. Sweetland, of Evans; Dr. Wm. A. Green, of Black Rock; Dr. Chas. A. Hyde, Lancaster; Dr. Jabez Allen, Aurora; Drs. Grove C. Gage and J. B. Pride, Alden; Dr. James Ives, Wales; Dr. Wm. Van Pelt, Williamsville; Drs. M. G. Lewis and Nathan Way, Black Rock; Dr. Josiah Barnes, a Yale graduate.

Dr. Austin Flint, already mentioned, came from Massachusetts in 1836, joined the medical society in 1841, was health physician in 1842, established the BUFFALO MEDICAL JOURNAL in 1845, and removed to New York in 1859.

Another physician to attract marked attention was Dr. James P. White, who studied with Dr. Trowbridge, and graduated in 1834. The establishment of the Buffalo Medical College, in 1846–1847, was largely due to his energy. He became the most noted obstetrician and gynecologist of his time, was the inventor of "White's obstetrical forceps," and the first to teach midwifery by practical or bedside demonstrations. He was one of the most active founders of the Buffalo Hospital of the Sisters of Charity, the Maternity and Foundling Hospital, the Providence Insane Asylum, the Buffalo General Hospital, and the Buffalo State Hospital. Besides the part he took in matters pertaining to his profession he was active in the Young Men's Association, the Academy of Fine Arts, the Historical Society and the Church Charity Foundation of the Episcopal Church.

Dr. Thomas F. Rochester, a member from 1854 to 1887, is too well remembered by the present members to need any special reference here.

The oldest living members of the society are: Dr. James B. Samo, who joined in 1844, after having practised here for four years. He was president of the society in 1862, and its librarian from 1852 to 1892—a period of forty years; Dr. John Hauenstein, a member since 1844; Drs. Louis P. Dayton and C. C. Wyckoff, since 1849; Dr. John Boardman, 1853; Dr. Edward Storck, 1854; Dr. George Abbott, 1855; Drs. Henry Nichell and F. F. Hoyer, 1857, Drs. John Cronyn and Leon F. Harvey, 1860; Drs. Thomas Lothrop and E. L. Bissell, 1861.

The presidents of the society since 1834 were as follows: 1834, Dr. Emmons; 1835, Dr. Alden Sprague; 1836, Dr. H. H.

Bissell; 1837, Dr. J. E. Hawley; 1838, Dr. M. Bristol; 1839, Dr. J. Trowbridge; 1840, Dr. E. Wallis; 1841, Dr. G. F. Pratt; 1842, Dr. Josiah Barnes; 1843, Dr. J. B. Pride; 1844, Dr. W. K. Scott; 1845, Dr. O. Wakely; 1846, Dr. F. L. Harris; 1847, Dr. Parsells; 1848, Dr. C. H. Austin; 1849, Dr. Wallace; 1850, Dr. Chas. H. Wilcox; 1851, Dr. A. S. Sprague; 1852, Dr. L. F. Ham; 1853, Dr. P. H. Strong; 1854, Dr. J. G. House; 1855, Dr. James P. White; 1856, Dr. Wm. Van Pelt; 1857, Dr. F. H. Hamilton; 1858, Dr. Austin Flint; 1859, Dr. L. P. Dayton; 1860, Dr. Wm. Treat; 1861, Dr. Sanford Eastman; 1862, Dr. J. B. Samo; 1863, Dr. Chas. Winne; 1864, Dr. C. C. Wyckoff; 1865, Dr. C. C. F. Gay; 1866, Dr. George Abbott; 1867, Dr. Thomas Lothrop; 1868, Dr. John Boardman; 1869, Dr. O. K. Parker; 1870, Dr. Julius F. Miner; 1871, Dr. Wm. Gould.

From 1872 the society's records are complete. The principal offices since then were held by the following gentlemen: 1872, president, Dr. William Ring; secretary, Dr. David E. Chace; treasurer, Dr. Wm. C. Phelps. 1873, president, Dr. Jabez Allen; secretary, Dr. Chace; treasurer, Dr. Phelps. 1874, president, Dr. Thos. Lothrop; secretary, Dr. Chace; treasurer, Dr. Phelps. 1875, president, Dr. John Cronyn; secretary, Dr. Chace; treasurer, Dr. Phelps. 1876, president, Dr. John Cronyn; secretary, Dr. D. W. Harrington; treasurer, Dr. Phelps. 1877, president, Dr. Henry Lapp; secretary, Dr. Harrington; treasurer, Dr. Phelps. 1878, president, Dr. Edward Storck; secretary, Dr. Harrington; treasurer, Dr. Phelps. 1879, president, Dr. S. F. Mixer; secretary, Dr. Harrington; treasurer, Dr. Phelps. 1880, president, Dr. F. F. Hoyer; secretary, Dr. Harrington; treasurer, Dr. Phelps. 1881, president, Dr. John Hauenstein; secretary, Dr. A. M. Barker; treasurer, Dr. F. W. Abbott. 1882, president, Dr. T. M. Johnson; secretary, Dr. Barker; treasurer, Dr. Abbott. 1883, president, Dr. S. E. S. H. Nott; secretary, Dr. Barker; treasurer, Dr. Abbott; 1884, president, Dr. J. C. Greene; secretary, Dr. Edward Clark; treasurer, Dr. Abbott. 1885, president, Dr. J. B. Andrews; secretary, Dr. Clark; treasurer, Dr. Abbott. 1886, president, Dr. E. T. Dorland; secretary, Dr. Wm. H. Thornton; treasurer, Dr. Abbott. 1887, president, Dr. O. C. Strong; secretary, Dr. Thornton; treasurer, Dr. Abbott. 1888, president, Dr. John D. Hill; secretary, Dr. Thornton; treasurer, Dr. Abbott. 1889, president, Dr. R. L. Banta; secretary, Dr. Thornton; treasurer, Dr. Abbott. 1890, president, Dr. G. W. McPherson; secretary, Dr. Thornton; treasurer, Dr. Abbott. 1891, president, Dr. E. C. W. O'Brien; secretary, Dr. Eli H. Long; treasurer, Dr. Abbott. 1892, president, Dr. Wm. Warren Potter; secretary, Dr. Long; treasurer, Dr. Edward Clark. 1893, president, Dr. John Parmenter; secretary, Dr. Long; treasurer, Dr. Clark. 1894, president, Dr. Wm. H. Gail; secretary, Dr. Franklin C. Gram; treasurer, Dr. Clark. 1895, president, Dr. Frederick W. Bartlett; secretary, Dr. Gram; treasurer, Dr. Clark. 1896, president, Dr. J. G. Thompson; secretary, Dr. Gram; treasurer, Dr. Clark.

The society now has 338 members.

